# Care That Matters: Classic Grounded Theory of Identity -Validation in Dementia Care

**DOI:** 10.1177/23333936261448366

**Published:** 2026-05-13

**Authors:** Judit Staller, Helene Hillborg, Annika Kjällman Alm, Lisbeth Kristiansen

**Affiliations:** 1 Mid Sweden University, Sundsvall, Sweden; 2 Region of Västernorrland, Sweden

**Keywords:** assistant nurses, behavioral and psychological symptoms of dementia, classic grounded theory, dementia care, person-centered care, Sweden

## Abstract

Behavioral and psychological symptoms of dementia in nursing care are complex and demand a highly professional approach. The study aimed to identify the main concerns and develop a classic grounded theory regarding assistant nurses’ care interactions when encountering these symptoms. Ten assistant nurses were observed during 70 hr of their shifts in two nursing homes. The *Theory of Validating Identity* was developed to maintain residents’ orientation, which became the core category. Life story, reminiscence, and other identified nursing care contexts, care interventions, and care actions demonstrate the smooth integration of person-centered dementia nursing care. *The Theory of Validating Identity* elucidates nursing interactions focused on care users’ identities and connected to two dimensions—body, mind, and soul—intertwined with components of time and space. Applying this theory to daily practice could optimize care management, facilitate the development of tailored, individualized care plans, and enhance clinical practice to foster advanced person-centered dementia care.

## Introduction

People living with dementia gradually experience a decline in cognitive, mental, and physical abilities, leading to a condition where their personal memory becomes limited. Moreover, comorbidities often accompany dementia (Skou et al., 2022). As a result, their interactions might merely reflect fragments of their past identities. (Fuchs, 2020). These factors exhibit care challenges for both formal and informal caregivers. Providing care to individuals with dementia in moments of confusion requires well-developed clinical skills and a person-centered approach. The Swedish eldercare strategy ensures that older adults receive comprehensive care in their homes as long as they desire, leading to a situation where the most cognitively and physically impaired older adults, who need round-the-clock care, often live in institutionalized nursing homes (Swedish National Board of Health and Welfare [Socialstyrelsen], 2022). The Swedish person-centered care philosophy and Kitwood’s model of person-centered dementia care emphasize the necessity of tailoring support to each individual’s unique experiences and resources. Both frameworks highlight the importance of mutual respect and a collaborative partnership between the carer and care user (Ebrahimi et al., 2021; Kitwood, 1997). While dementia could be perceived as distorting one’s sense of identity, research indicates that self-perception persists even in care users with severe dementia (Álvarez-Fernández & Marín-Carmona, 2024; Batra et al., 2016). Consequently, preserving personhood is important for understanding the person during care challenges, which in turn assists in providing more suitable, personalized caregiving.

Each year, between 20,000 and 25,000 individuals are diagnosed with dementia in Sweden, with the social cost of dementia exceeding SEK 100 billion annually (Frisell et al., 2023), and it is estimated that approximately 90% of people with dementia have some form of behavioral and psychological symptoms of dementia (BPSD) over the lifespan of the disease (Devshi et al., 2015). Research suggests that 30% to 50% of people living with dementia in community-dwelling settings suffer from at least one type of BPSD, which includes a range of behavioral symptoms such as aggression, wandering, restlessness, and apathy, as well as psychological symptoms like depression, anxiety, and hallucinations. (Cloak et al., 2025; Kwon & Lee, 2021). Encountering BPSD in diverse healthcare settings challenges formal caregivers and therefore increases reliance on psychopharmaceuticals. However, non-pharmacological, person-centered approaches can reduce symptoms in a nursing professional manner, avoiding the adverse side effects of medicalizing BPSD, while improving the well-being of both individuals and caregivers (Alm et al., 2018; Meng et al., 2021).

In Swedish social care institutions, assistant nurses (ANs) are the most frequent bedside care providers. They are often delegated clinical responsibilities, such as administering oral medication, performing catheterization, and conducting blood sampling. Supported by 2 years of formal vocational health education, ANs perform both practical care tasks and essential nursing duties. The government has awarded protected professional titles to recognize the ANs’ essential bedside care work and to acknowledge the importance of their high-quality care and patient safety. A protected professional title is a legal status that reserves the use of a particular health profession for those who meet predefined qualifications or certifications. Furthermore, ANs are the primary social and healthcare professional group in Sweden, accounting for 51% of Sweden’s local government health and social care staff (Swedish National Board of Health and Welfare [Socialstyrelsen], 2024a).

A holistic approach to person-centered care helps preserve a sense of personhood and successfully manage BPSD by integrating the person’s identity into nursing care, while the self receives a personal context of the entity (O’Donnell et al., 2022). Empathetic care, within the framework of person-centered care, enables nurses to identify factors that influence and uphold an individual’s sense of identity and maintain it through individualized interactions tailored to meet each patient’s specific needs (Rose & Dening, 2023). Consequently, nurses play a crucial role in enhancing self-awareness, which, in turn, improves the quality of life, autonomy, and mental well-being of individuals with dementia (McCormack et al., 2012).

Advanced dementia tools such as life story work and reminiscence therapy have proven helpful in dementia care by affirming care users’ memories from the past and thereby effectively addressing BPSD. Life story work utilizes narratives and facts from the individual’s life to improve care actions, while reminiscence therapy evokes memories, knowledge, and emotions to maintain cognitive function. (Butler, 1963; Grøndahl et al., 2017; Park et al., 2019). Within the framework of person-centered care, reminiscence and life story correlate with better health outcomes and higher quality of life among people with dementia, and they have a positive effect on the prevention of BPSD (Cooney & O’Shea, 2019; Woods et al., 2018).

In Sweden, the use of life stories to care for people with dementia is recommended by the Swedish National Board of Health and Welfare (Socialstyrelsen, 2024b). Digital care tools, such as digitalized life stories, enhance dementia care by providing an interactive platform for sharing personal experiences and important life moments, benefiting both residents and caregivers (Dellkvist et al., 2024). However, the applied life story templates often fail to support caregivers effectively, as there is no coherent methodological approach to determine which narratives and facts are significant, nor is there guidance on how to incorporate them into daily care (Möllergren & Harnett, 2024).

BPSD creates significant challenges to the successful implementation of person-centered care, ultimately reducing the well-being of both residents and nurses in nursing homes. There is still much to learn about how nurses manage the challenges of BPSD care during their clinical work. Observing the care delivery can help identify what actually happens during the care process, thereby enabling a more nuanced conceptual understanding and further implications that can inform education. The knowledge gained from such observations not only deepens understanding of care practices but also contributes to more competent, responsive care. This, in turn, advances dementia nursing, promotes higher-quality person-centered dementia care, and equips formal caregivers with theoretical insights that can be embedded in practical nursing tools and interventions.

## Objective

The study aimed to identify the primary concerns of assistant nurses regarding nursing care interactions in nursing homes when caring for individuals with BPSD, subsequently developing a Glaserian-classic grounded theory (CGT) to explain these processes.

## Method and Methodology

CGT is a method that elevates everyday research on social behavior to a more abstract level, generating a theory that describes and explains the interrelationships among social patterns and behavior. CGT research is well-suited for exploring emerging ideas in unfamiliar contexts or for re-examining established knowledge, allowing familiar concepts to be interpreted from a new perspective. We employed CGT, as developed by Glaser (2001), to generate a conceptual theory grounded in empirical data that demonstrates ANs’ care interactions. The CGT method helped us to internalize new conceptual understandings of ANs’ interactions in person-centered dementia care, enriching and expanding existing nursing care knowledge and practice. This methodological approach was particularly suited to our data collection technique, observations of everyday nursing care interactions between ANs and residents in dementia care. By focusing on observed behavior rather than descriptive accounts, CGT illuminated the latent social processes underlying ANs’ interpersonal interactions as they managed care. The methodology’s philosophical orientation—to conceptualize behavioral patterns rather than describe behaviors—aligned with the study’s aim to uncover what was actually happening during caregiving.

## Settings and Participants

The study was conducted across two nursing homes in a smaller city in northern Sweden, with a regional population of approximately 245,000. The Head of Eldercare in the municipality was asked to approve the research and select appropriate nursing homes that met the study’s inclusion criteria. The inclusion criteria specified that the selected care units primarily served residents with dementia and that the ANs had extensive experience caring for individuals with dementia. However, no formal educational qualifications were required for dementia care. The operations managers determined the ANs’ experience. The research team met on-site with operations managers and ANs from four units in two nursing homes to explain the study’s objectives and design. More than 10 ANs volunteered. The number of participating ANs varied across units, with an uneven distribution. All observed ANs were women. A schedule was arranged for 10 ANs, including dates and shifts. First author was engaged with the data collection and analysis during the observations. In total, 70 hr of observation were completed between 7:00 AM and 9:00 PM. Ten ANs were shadowed, and each AN was observed for approximately 4 hr on two separate occasions during their duty. Altogether, approximately 20 residents were cared for during the observations. After the 10th AN, no additional observations were needed, as the data demonstrated saturation. Data were collected from February to April 2024.

### Data Collection and Analysis

The exploratory design of CGT commenced with open coding and categorization to uncover variation and patterns in the data, followed by theoretical coding guided by the researcher’s theoretical sensitivity (Glaser, 1978). Throughout the analysis, the constant comparative method was employed to refine codes and categories, identify latent patterns of behavior in the data, and integrate these conceptually into a developing theory. During both stages of open and theoretical coding, codes were generated incident-by-incident and were continuously compared with codes from memos. This iterative analytic process enabled the continual refinement and integration of emerging concepts into a coherent theoretical framework (Glaser, 1998). The step-by-step description of the method and outcomes is shown in Table 1.

**Table 1.** Steps and Outcomes of the Data Analysis.*

No.	Data analysis		Outcomes
1	Spradley’s matrix	Using it as a template to collect data	Maximize data acquisition
2	Parallel data collection and analysis	After each observation, field notes were immediately analyzed	Initial coding
3	Open coding and categorization	Writing and organizing open codes commencing substantive categories	Everything was data during this phase.
4	Memo writing	Simultaneously throughout the whole research process, the first author wrote extensive memos about ideas and thoughts that contributed to analytical thinking.	In the beginning, for the emergence of ideas, and later for the conceptualization and integration of relationships
5	Constant comparison method	Throughout the whole research, codes and categories were continuously compared and tested, along with concepts of memos, to reach an abstract level.	To understand what the ANs were striving for
6	Discover the main concern.	The main concern shows the actual problem	The main concern: *Not triggering further manifestation of BPSD*
7	The core category is emerging	The pattern and the latent behavior are revealing	The core category provides the answer to the “main concern.”
8	Core category identification	The core category addressed the main concerns about ANs’ behavior during their care interactions.	Core category: *Maintaining orientation in nursing care*
9	Theoretical coding and categorization	Through the constant comparative method, analysis of the latent patterns linking the core category and its related categories facilitated the theoretical integration of codes, categories, and concepts.	Identification of properties, subcategories, and categories. The two categories, which are caring contexts: Awakening Identity and Shifting Orientation,
10	Integration	Finding the conceptual pattern and relationships between the variables helping leverage the theoretical framework	Subcategories and their properties occur and are named as subcategories: cate interventions, properties: care actions
11	Theoretical saturation	No more properties emphasize the theory	The contours of the theory become visible
12	Theory development	The theory emerges through the iterative sorting and comparison of codes, categories, and memos, gradually forming a framework that integrates all conceptual elements.	*The Theory of Validating Identity* was born as a framework that reveals how ANs interact with residents to maintain their orientation during nursing care

* The table summarizes the interwoven and parallel analytical processes characteristic of CGT, complementing the method of analysis.

In line with the CGT’s exploratory research, particularly during the open categorization phase, it was significant to focus on “What is actually happening in the data?” and “What is the main concern of the participants?” (Glaser, 1992). The research team acknowledged that Spradley’s participant observation matrix for cultural domains and semantic connections would encourage broad, inductive observation without relying on any pre-imposed concept. A pilot observation was conducted before data collection, in collaboration with LK, a senior researcher with extensive experience, to verify whether the matrix indeed facilitated broad data collection. After pilot observation, Spradley’s nine ethnographic dimension matrix that accounts for: space, actor, activity, object, act, event, time, goal, and feeling—demonstrated a flexible analytical guide for writing field notes and memos that considered various aspects of the observed social situation, providing a natural entry into CGT’s open coding and categorization process (Table 1, item 1).

The first author continued collecting and analyzing data in parallel, along with memo writing, following CGT’s approach, which highlighted the simultaneous and comparative nature of data collection and analysis. During the empirical research, the team held frequent discussions (Table 1, items 2 and 4). When natural pauses occurred during observed care work, the researcher asked follow-up questions to clarify social interactions. The responses were recorded alongside the field notes. Throughout the open coding phase, the researcher consistently asked: What is this data a study for? (Glaser) What is actually happening? (Table 1, item 3). The constant comparative method helped the observer identify the study’s main concern (Table 1, items 5–6).

After the main concern identification, the open coding and categorization process was gradually replaced by the theoretical coding and categorization process. In this phase, interpretations and hypotheses were continually tested and refined through Glaser’s method of constant comparison of codes, categories, and concepts from memos, contributing to conceptual thinking and elucidation of the meaning of these concepts, leading to the identification of the core category (as shown in Table 1, items 7–9). Once the core category, which addressed the main concerns of ANs’ social behavior, was identified, the relationship among the core category’s variables emerged (Table 1, item 10). When additional data revealed no further relationships during the conceptualization process, the data collection and analysis were considered saturated (Table 1, items 10–11). The CGT’s constant comparative method resulted in a substantive theory that was grounded in its related variables and further developed this result at an abstract level (Glaser & Strauss, 2017; Table 1, item 12).

### Ethical Approval and Consideration

Several steps were taken to protect residents involved in these observations, based on the principle that avoiding exploitation is fundamental in academic research, particularly when participants belong to vulnerable or marginalized groups, including individuals with cognitive impairments. During observations, this principle was protected not only by the observer-researcher but also by the observed ANs, who demonstrated profound commitment to ethical principles and continuously endeavored to safeguard residents’ dignity and privacy. Each observed AN explained the researcher’s presence to both the residents and relatives (The World Medical Association, 2024). Due to fundamental ethical considerations, such as respect for self-determination and the intent to avoid marginalizing or overburdening a vulnerable population, the direct perspective of people living with dementia has been absent from this study. During the research, a sign was posted at the nursing homes’ entrances to inform healthcare personnel and visitors that an observation was in progress. Verbal and written information about the study was provided to the operations managers and the volunteer ANs, who all signed the information letters and consent forms, which were kept in a double-lock container (ALLEA, 2023; The World Medical Association, 2024). Before each observation, the staff who worked during the shift approved their consent verbally and in writing. This was in accordance with the (SFS 2003:460).

The Swedish Ethical Review Authority approved this study (registration number 2023-03560-01).

## Result

**Figure 1.**
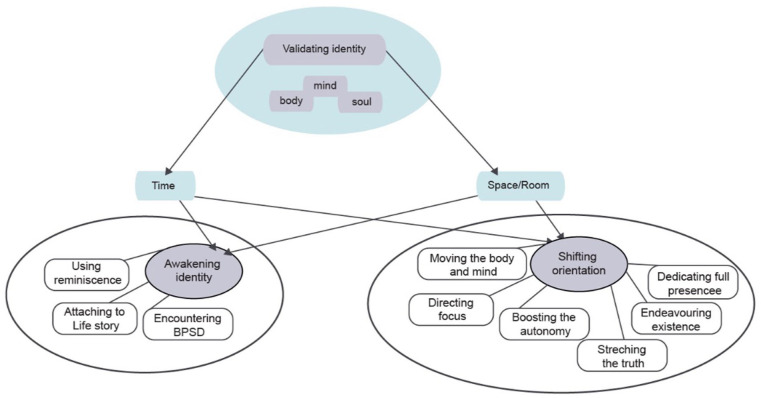
The theory of validating identity.

The ANs’ main concern was “*Not triggering further BPSD manifestations*,” and the AN addressed this concern by “*Maintaining orientation during nursing care*,” which became the core category. Whenever residents appeared confused, ANs perceived that the residents experienced disconnection from time and space. On these occasions, ANs moved beyond care, using care interactions as opportunities to orient residents. Delivering adequate nursing care underpinned by ANs’ main concerns reassured residents’ sense of orientation in time and space. Based on the core category, the theory was developed to illustrate the relationships of the identified ANs’ care actions. The *Theory of Validating Identity* emerged, accentuating how ANs resolved their main concern when encountering a person with dementia suffering from BPSD. The theory illustrates how ANs validated residents’ identities, understood as the combination of body, mind, and soul, by integrating time and space to reorient them during moments of confusion and identity loss. Time is the boundless, continuous extension in which nursing interactions occur and in which positions and directions are used. Space is a dimension of the theory that manifests in different rooms in the nursing homes. Time and space constitute intricately interconnected components of the theory’s conceptual framework, integrating targeted nursing care interactions. Figure 1 illustrates the theory, its dimensions, and interconnections.

**Figure 2.**
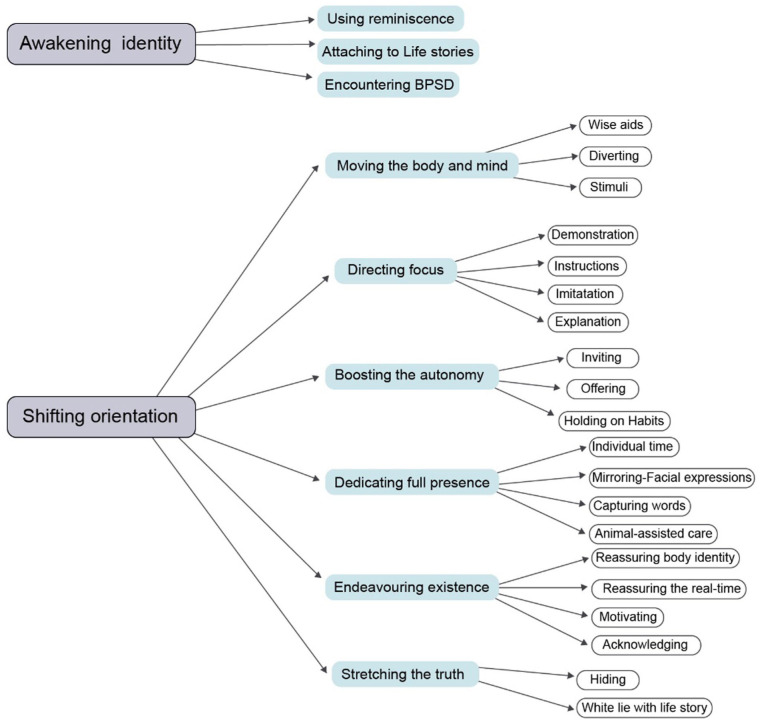
Care contexts, care interventions and care actions.

*The Theory of Validating Identity* is built on two main care strategies: Awakening identity and Shifting orientation. The theory’s time and space dimensions intertwine with these two extended care strategies, which embody several care actions, and symbolize affirmation of body, mind, and soul. In the *Theory of Validating Identity*, time is not a linear progression, but rather a fusion of the past, present, and future, in which they intermingle. ANs’ care actions often focus on reframing the perception of time by reorienting residents’ identities when interacting with them. Similarly, space is not limited to a single location; rather, it comprises various areas within the nursing homes, including residents’ rooms, the living room, corridors, and the dining room, each providing a unique set of cues for awareness and sensory experience. Several care actions and their connections to the care strategies are illustrated in Figure 2.

### Awakening Identity

The caring context of awakening identity elucidates ANs’ effort to shift the focus between the present and the past to evoke a broader range of positive emotions. This care context illuminates the following care interventions: using reminiscence, attachment to life stories, and encountering behavioral and psychological symptoms of dementia.

#### Using Reminiscence

This care intervention is rooted in the past, evoking feelings, perceptions, and experiences of times gone by. Reminiscence as a feeling was initiated by the observed ANs, and is rarely started by the residents. Reminiscence involves the recollection of sensations across all senses, such as sight, sound, taste, smell, and touch. It encompassed, for instance, from the observed old objects, photographs, or films that ANs present to the residents—listening to nostalgic hits or the gentle singing of artificial bird toys; enjoying coffee with biscuits or chewing candies; inhaling the aroma of food or blooming flowers in the garden; feeling objects, caressing the skin, and petting an animal. Emotions were observed during nostalgic experiences, such as when residents sat on a balcony at sunset while ANs grilled sausages. Within reminiscence care drew on Swedish culture and traditions to support a culturally framed sense of self. Many observed nostalgic but artificial items, such as cuddly toys, artificial flowers, or vintage toy cars, were not authentic but could evoke feelings, conjuring perceptions of bygone eras and answering the question: “Who am I? I am my memories and my emotions.” Coffee with cake was viewed as a cultural practice where drinking coffee is more than just a beverage; it is tied to perceptions of Swedish coffee-drinking culture. Specific sensory prompts were observed, including the use of pleasant tastes—such as yoghurt, chocolate, or candies as pre- or post-meal cues, as well as sensory stimuli such as the scent of a live dog.

#### Life Stories

Life stories served as a valuable care intervention for uncovering residents’ identities. They evoked personal memories and individual experiences through personal narratives. This strengthened connections between the present and positive experiences in the past. These stories helped people with dementia recognize the significance of time and space, ultimately aiding their understanding of the key milestones that shaped their identity. Knowing from life stories that the residents liked singing and dancing, the observed ANs could utilize these as care instruments in their care activities. Fragments of life stories ultimately fostered well-being by affirming the person through positive memories of personal history. During observations, ANs used concrete cues to prompt memories and start conversation: photo prompts (e.g., a cat on the wall): “*Are you thinking about the cat?*” “*Do you remember your cats?*” Every day anchors: “*Have you picked a lot of blueberries when you had a garden?*” (AN 6). While life stories mobilized ANs with context regarding a resident’s preferences, they often failed to explain why the triggers for anger, which could have been related to the cause of BPSD, for example, when something happened in the resident’s life that evoked bad memories during care. The essential aspects of life stories seemed to rely on the content of the depth of personal details provided by family members in the life story template before the resident entered the institution, and these documents were filed with the resident’s care documents. The life story symbolized the “real” identity, as the AN said during a follow-up interview: “*We should not see them* (residents) *as they are now but as they used to be*” (AN1). “Life stories in reverse” also occurred during observed caregiving. ANs observed while they were sharing fragments from their lives when they wanted to commence a conversation during caregiving. This confirmed the present moment and established a connection or redirected attention from negative emotions, as in the observed dialogue: “*Do you remember when I put salt in porridge instead of sugar*?” (AN5).

#### Encountering Behavioral and Psychological Symptoms of Dementia

This care intervention explored how ANs influenced residents’ perceptions of the past. When residents struggled to express their feelings, frustration could arise, leading to BPSD. ANs strived not to confirm negative feelings but rather to find care solutions to help residents leave behind feelings tied to negative memories and orient them to the present. When the observed AN encountered BPSD, they concentrated on the resident and avoided multitasking.

It was exhausting to deal with anxiety constantly. When the ANs were observed exhausted, they temporarily withdrew their help, reassuring the resident that they would return. Before they left, they ensured the environment was safe for the resident to be left alone. They tried to remain calm to prevent the escalation of negative feelings and avoid coercion. If this approach was ineffective, ANs stepped back to allow the resident to be alone, then returned later with a new strategy. According to observations, in response to BPSD, ANs remained calm and attentive, endeavoring to prevent escalation. If the efforts were insufficient, they sometimes temporarily withdrew to give residents space, reassuring them with phrases such as “*See you in a while!*” (AN 7).

Trauma could be detected among BPSD, even if it was not mentioned in life story templates. ANs could not prevent residents from continually re-experiencing past trauma through intense emotions. In such cases, ANs avoided reminiscences or life stories that directed residents to the past; instead, they used their presence and body contact, offering a hand for residents to hold, to help feeling they were not alone in real time. During observation, this was observed: “*Look at me. I am here with you right now*” (AN 7). Additionally, the observed objects and environments, such as soothing music, cuddly toys, and a relaxing atmosphere, shifted attention and reduced distress during BPSD. Dealing with BPSD required ongoing attention and deep focus. Consequently, these purposefully arranged calming settings were essential for ANs as well, helping them manage their own emotions during care work.

### Shifting Orientation

Shifting orientation illustrates several care interventions and associated care actions that help escorted residents return to a suitable time and space during the confusion phase. The greater number of interventions portrays the complexities of this caring context and the diverse selection of AN’s skills in finding multiple care solutions during BPSD. This caring context illuminates the following care interventions: Moving the body and mind, Directing focus, Boosting autonomy, Dedicating full presence, Endeavoring existence, and Stretching the truth.

*Moving the body and mind* demonstrated how ANs moved residents’ bodies and minds during care interactions. This care demanded AN’s full attention, maintaining the real-time dimension while adjusting the space. Its three care actions are: wise aids, diverting, and stimuli. Wise aids were widely implemented in the observed Swedish nursing homes, utilizing automated, robotized, and digitalized welfare technologies. Various modern therapeutic aids were available to care users, such as cuddly singing birds that could induce perceptions and stimulate the senses when pushed; a dance vest that functioned as a biofeedback tool, pausing the music if the resident stopped dancing to encourage physical activity and body awareness. Additionally, a projector offered different exercises designed to stimulate cognitive function alongside arm and hand coordination. Some observed ANs used their private smartphone applications to create playlists of old Swedish hits from the 1950 to 1960s and played them when they judged it suited to calm the care users. During observations, there were also staff aids that helped with time and body orientation, such as smart devices like continuous glucose monitors, as well as lifts and turners that assisted with transferring residents’ bodies from one room to another or lifting them to make the bed. Diverting: This care action moved the resident’s mind and body to the present, for instance, when the observed AN shared a reminiscence from their life story, thereby leading the mind away from unpleasant situations. Mentioning family members’ names and activities could distract residents’ attention and alter their mood. Observed dialogues that demonstrated concrete diversions: “*Come with me*,” “*Are you going to rest now?*” “*Let’s stay here and have coffee*,” “*Some morning news?*,” “*Are you in the mood for coffee?*” Task aimed diversions were observed when the residents were asked to accompany AN to fold laundry, fetch milk from the basement, or go for a short walk with AN’s dog outdoors. The observed AN diverted an unpleasant care with an actual or made-up narrative: “*I almost hit a fox with my car yesterday*” (AN 6). Stimuli were observed while ANs were telling diverse story fragments from the past, reading aloud a story, or singing and reciting rhymes and proverbs from the residents’ generation period, which provided sensory or cognitive stimuli. Additionally, the observed activities, such as afternoon movie time, offered stimulus for reminiscence by watching old movies. The observed spa day, which included massage, nail polish, and makeup created by ANs, provided a relaxing, sensory experience for the residents. *Directing the residents’ focus* involved organizing care actions to help gain recognition and alleviate confusion. This was achieved by linking care to the residents’ awareness of the present moment and by supporting their understanding of the ongoing care activities. The four care actions are demonstration, instruction, imitation, and explanation.

The demonstration showed that ANs’ interactions confirmed the time of day, as observed in preparing the room for bedtime and tidying it for the morning or evening routine. Telling what to do while demonstrating the activity placed the identity in time and place, like during the observation: “*This is your room! Here, we will enter the bathroom*” (AN 1, 6, 7). ANs were observed using instructions to support residents’ understanding and to deliver care in real time, saying, “*Extend the leg!*” *Open your mouth!*” (AN 1, 3, 4, 6). These actions maintained residents’ ability to carry out tasks independently. Instructions could also remind the resident to do something that could lead to a future need, like in the observed conversation: “*Tell me if you need to go to the toilet*” (AN 2). Imitation referred to a care action that functioned as a supplement to instruction confirming the verbal request. The observed ANs often stressed demonstrating how to brush teeth, imitating the act of swallowing medicine, or even showing how to sit on the sofa and watch TV. Explanation occurred when ANs tried to interpret an incident, clarifying what was happening and reassuring residents that anxiety was unnecessary. For example, the observed resident wanted to pay for coffee, and the AN answered: “*Don’t worry, it is included in the rent*” (AN 7). When this care action was observed during other care settings, the following dialogue was noted: “*You are here because you need help. We are here 24/7 to help you. There is nothing to worry about*” (AN 6, 9). This is how supporting resident awareness and understanding are associated with everyday activities and are linked to the present time and space.

*Boosting autonomy* counterbalances the resident’s subordinate position in nursing homes. During observations, ANs pursued guarding freedom of choice, a hallmark of sustaining autonomy in identity. No coercion was permitted during care, as forcing residents is strictly forbidden under Swedish nursing ethics and could also cause greater confusion. The three care actions are inviting, offering, and holding on to habits. Inviting involved the residents confirming their permissions to ANs. This care action encouraged the residents to either accept or decline an offer, emphasizing the human principle of freedom of choice; it was not always applicable in care. During observations, inviting residents to an activity sometimes remained just a formality, as the present lacked meaning for residents, as it was in the observed dialogue: “*Can I get you a cup of coffee?* (AN 1, 2, 5, 6, 10). *Do you want a sandwich or porridge?*” (AN 3, 4, 6). In these instances, the residents remained silent and did not provide permission. During the care action: offering, ANs were observed seeking residents’ consent and making the necessary arrangements in accordance with their wishes. Requesting permission to act was similar to inviting someone to provide a service or care, and to gain mutual consent, as in the following observed conversation: “*I am not the one who decides what you prefer*” (AN 1). “*Is it okay if I cut your nails?*” (AN 2). Holding on to habits was a vital care action that adjusts daycare activities and nursing care according to the residents’ specific needs, rather than strictly adhering to the institutional timetable. There were significant opportunities to adapt care and activities according to the residents’ previous habits. For instance, it was observed that care could be adjusted for those who prefer to sleep later in the mornings or rest later in the evenings. Some care customs aimed to distinguish working days from weekends, such as the practice of serving dishes on a fine dinner set on Sunday, a Swedish cultural tradition that stresses the importance of distinguishing Sundays from working days. Additional individual customs and habits had been carried over from their earlier adulthood and are documented in their written life stories.

*Dedicating full presence* signifies sustained care when time is devoted to companionship. Caregivers allocate time for focused attention during these care interventions, enhancing residents’ identities and validating their emotions. This care intervention had four care actions: individual time, mirroring facial expressions, captivating words, and animal-assisted care.

Individual time implied being present with both body and mind for the sake of security in care and creating an intimate relationship. This care action was framed as a form of companionship that builds awareness and can be especially important during personal hygiene and care. This can be critical when multiple safety measures are needed to prevent harm when leaving the resident alone. For example, it was observed that when an AN sat down while administering medicine, the resident received complete focus from the ANs. Eye-to-eye contact emphasized attention in the present moment, a moment of dedication to the person without authority, affirming the individual’s freedom. This action was also used when the observed AN dedicated time to encourage calmness and full concentration in real time during BPSD encounters. The resident received personal dedication as they were in the center of the dedication. An example was when the observed AN said, humorously, “*Look what service you have!*” (AN1). Mirroring facial expressions was used when ANs provided feedback in the present and maintained emotional orientation. This could be a smile, like during observation when the AN affirmed the resident not to worry: “*Do not worry, I am helping you*. (AN 5). *I am here with you*” (AN 8). A worried face mirrored the confirmation, like when AN expressed: *I understand your worry and bad feelings*” (AN 3). Additional conversations were heard during the observation when an attempt was made to see a smile on the resident’s face as an affirmation that things were going well: “*We are good, both of us*” (AN10); “*You are so beautiful!*” (AN7); “*What a handsome man!*” (AN3). Capturing words care action was used when the resident said something that neither the resident nor the AN understood the meaning or reason for. Repeating phrases by the ANs brought joy and humor to their interactions because it reflected their intention to interpret and find meaning in the residents’ words and sentences. Sometimes this led to misunderstandings, but those moments surfaced memories on both sides and encouraged mutual communication. For example, during observation, a conversation turned into the AN’s playful repetition of words, but the meaning remained unclear for both of them. This sparked shared humor, probably triggering memories from both parties. A shared laugh confirmed a pseudo-understanding. During the observation, the resident said “*Thanks*,” and the AN tried to understand the meaning behind it. However, the AN couldn’t understand the reason, and the residents might have already forgotten what they wanted to say. Animal-assisted care triggered real-time moments that evoke positive emotions among residents. This is attributed to the appreciation for animals, who do not perceive the past or future but only experience the present, reaffirming mutual existence. A live, domesticated animal draws attention to the smell, the mimics, and the fur of the animal, making the present moment and feelings real. Not only are living animals used, but helping during care by using a plush cat when the resident is scared creates a calming, present-focused moment.

*Endeavoring existence* care intervention means strengthening one’s identity in the present moment, in mind and body, during the care process. It involves four care interactions: reassuring body identity, reassuring real-time, motivating, and acknowledging. Reassuring body identity occurred when ANs provided personal hygiene care. During this time, ANs and the residents were in an intimate relationship. Here, AN created a tight, intimate connection while attempting to confirm body integrity in real time. The following examples were heard (as due to the dignity, the observer did not observe) during this care action: “*I see that your nails are long*. (AN 10). *You have some skin flaps in your mouth*” (AN 2); *Your lips are a bit dry* (AN1). Reassuring real-time occurred when the time of day was indicated during care. During observation, the following sentences were observed: “*Good night, sleep well!* (AN 10). “*It is lunchtime now*” (AN 3). A specific time could also be distinguished, like when one of the observed AN said, “*Today is a good day! Today is the 1st of March!*” (AN 5). AN could also confirm the weather in real time when it was heard: “*It is snowing and very cold*” (AN 10). This care action was also suitable for signaling, for example, that it was morning or when it was dark in the morning and afternoon, or the date of the year. For example, it was observed that when AN wanted to signal the morning through the environment, she opened the window blinds, made the bed, and arranged the room; named the day or date; or even affirmed seasonal traditions and indicated future activities on that day. One observed nurse said, “*There will be waffles served today*” (AN9). Care action could also be used for reassurance of the weather or place: “*It is snowing and very cold*,” “*We must stay inside*” (AN8), and was even observed with the intention of situational anchoring: “*We should stay here and have a coffee*” (AN8). Motivating was used when the aim was to praise the resident for something that would, in turn, reaffirm the resident and confirm the resident’s existence in the present. Observed AN said: “*I am so impressed with you!*” (AN 3). Other times, AN encouraged residents by saying, “*We take your pace! You can do it yourself!*” (AN 4). These sentences endorsed the residents’ ability to perform activities without instructions. Acknowledging was a care action that assured the present, helping ANs confirm that care was being delivered pleasantly from the resident’s perspective, as when AN was observed asking, “*Are you satisfied?*” (AN 10) “*Is it going well?*” (AN 2).

*Stretching the truth* refers to how some ANs might feel compelled to deceive residents, often by hiding information or telling white lies with life stories. Hiding information was employed to secure care and healthcare. During observation, AN adapted the food to make it easier for residents to chew and swallow medication. Hiding medicine in the food could mislead the residents’ body and mind orientation. For example, when the observed AN administered the medication by mixing it into yogurt without informing the residents, or when the AN wanted to ensure that the residents would swallow it, the resident was served yogurt instead of the requested sandwich, thereby misleading them. Generally, food was modified to be easy to swallow, which could be deceptive, as its appearance and texture might not be appealing to residents. White lies with life stories were bits of information from life stories that are used to calm BPSD issues. These stories were fabricated, tied to the person’s life history, and woven into fictional narratives. From the follow-up conversation, ANs reported feeling forced to lie when family members hid the truth from their relatives, leaving them with no choice but to continue lying. For example, when an observed female care user became confused, dressed up, and wanted to leave the nursing home, the observed conversation was: “*Where is my husband? (Resident)*” “*Your husband is with your son at the cottage*” (husband died several years ago, and family members did not want to tell her; AN 7). ANs saw this care action as ethically challenging but felt they had no choice but to keep maintaining the constructed reality and continue engaging this narrative in care. Furthermore, white lies could be used by ANs’ own initiative to dispel worries, like in the observed situation when the resident asked AN whether they had a car to travel home, and the AN answered: “*There is no bus tonight; you must sleep here tonight. I will give you a lift home tomorrow*” (AN 5). The intention of using white lies was good, since bringing pleasant, positive information from the past into the present could have alleviated confusion. However, residents became more confused when they sensed that something was not right. Another conversation during observation was also noted when one resident constantly wanted to go home, and the AN informed her that there had been water damage at her house and advised her to wait until it was fixed. Unfortunately, this care action had been based on a white lie intended to motivate her to move out of her home due to her extensive care needs. Since them this lie could never been changed, and it slowly, so thoroughly, covered reality that the resident doubted her own perception and the truth. In real time, the house had been sold; there was no way to return to reality anymore. As the conversation between the AN and the resident continued, the resident was asked whether she had had a dream during the night. She explained that in her dreams, she longed to go home and thought, “*But I don’t know how to get home.*” Often, the need and use of white lies were not documented in the resident’s care documents. Still, every AN knew all the false information about all residents and actively used it during care interactions. Lies did not help residents orient themselves in place and time; instead, this misinformation increased residents’ BPSD.

## Discussion

Using classic grounded theory, the study sought to identify ANs’ main concerns during care interactions with residents experiencing BPSD in nursing homes. Analysis indicated that ANs’ main concern was preventing the escalation of BPSD manifestations, which was named: “*Not triggering further BPSD manifestations.*” This concern emerged from the data and was resolved through the core category, “*Maintaining orientations in nursing care*,” which explains how ANs continually address their main concern in practice. By orienting residents, ANs demonstrated their attempt to sustain stability by affirming residents’ identities during nursing care. The theory elucidates how ANs address residents’ negative emotions and mood disturbances by stimulating the body, mind, and soul within the specific context of time and space during their care interactions. The developed *Theory of Validating Identity* accounts for two caring contexts—Awakening identity and Shifting orientation—and their associated variables demonstrate how therapeutic approaches, commonly used in group interventions such as life stories and reminiscences, are interpreted and adapted differently in nursing care, particularly when supporting residents experiencing BPSD during person-centered care.

The theory encompasses several ways life stories are applied in care. These insights can guide the development of life story templates and care plans. The theory extends this by exploring insights into how nursing staff navigate reminiscence in care interactions, influenced by deeply rooted cultural beliefs and traditions, revealing that reminiscence is not just a group therapy method but also a vital nursing tool that can be seamlessly integrated into everyday nursing interactions with individuals living with dementia.

The *Theory of Validating Identity* explains a recurring pattern in which nursing actions are adjusted to residents’ life stories and experiences. It also accounts for ANs’ use of limited self-disclosure, described as a “reverse life story,” through which ANs share brief personal stories to establish connection during caregiving. This pattern facilitates reciprocal interaction and redirects residents’ attention away from distress during periods of confusion. Through these exchanges of personal stories, identity is reinforced via shared positive memories grounded in individual histories, supporting person-centered caregiving. In contrast, the care action “white lies with life stories” disoriented the residents, creating more confusion. Dishonesty in sharing life stories risks undermining trust and destabilizing the residents’ sense of self, leaving them more suspicious, unsettled, and unsure of what is actually happening. During BPSD, the misuse of life stories in nursing practice can further confuse residents and fail to provide them with a clear sense of orientation to time and space. In this instance, the *Theory of Validating Identity* does not apply. The two caring contexts, along with their associated care interventions and care actions, illustrate how ANs strive to maintain residents’ orientation through separate and individually chosen nursing care. These identified care strategies operate within the dimensions of body, mind, and soul, interconnecting and intertwining different aspects of time and space when confusion arises.

The *Theory of Validating Identity* aligns with prior work on personhood, which highlights the qualities and traits that constitute an individual’s existence (Hennelly et al., 2021; Jenkins & Price, 1996), extending understanding of how these dimensions are enacted and maintained in nursing care. Identity includes aspects that define a person and shape their sense of self in time. Additionally, identity is not solely an internal construct; rather, it is socially influenced and reflected through interactions with others (Locke, 2004). The social dimension of identity is central to Kitwood’s theory of person-centered care, which emphasizes the close link between personhood and well-being. A person’s sense of identity is shaped not only by their perceptions of personhood but also by how others acknowledge and reflect their identity to them (Kitwood & Bredin, 1992; Mead, 1913). Therefore, developed and adequate nursing care is particularly relevant in dementia care, where people with dementia often experience fragmented or shifting self-perception (McCormack et al., 2012). Through competent care, ANs serve as mirrors that reinforce residents’ identities, making their role crucial to residents’ self-perception. The *Theory of Validating Identity* explains how ANs enhance personalized care when the progression of the syndrome is moderated by supportive intervention and a nurturing environment. By eliciting positive emotions and modifying the dimensions of time and space, ANs actively reinforce and transform residents’ identities.

Personhood is a dynamic attribute of Kitwood’s person-centered care (Kitwood & Bredin, 1992), and has the capacity to develop over time, suggesting that it can be understood as a flow of energy that continually rises to the surface and is more spiritual than cognitive in nature (Tolhurst et al., 2017). Consequently, dementia nursing support becomes more temporally and energetically efficient when ANs can carry out their nursing tasks in a way that aligns with the residents’ current state, rather than requiring their full, conscious attention and participation. However, in this case, the residents would gradually lose their competence to maintain functional and cognitive ability, and their health condition would deteriorate faster, especially when they are mentally confused. In this way, they would also lose their ability to maintain their sense of identity.

The capability approach aligns with the pursuit of dementia nursing that strives to emphasize identity affirmation, especially in mood disturbances and confusion (Diener, 1984; Johansson et al., 2022). While encountering BPSD, ANs endeavor to preserve the resident’s autonomy and compensate for the loss of capacity, which is associated with care users’ life satisfaction. Despite cognitive and emotional confusion, the resident’s sense of self can be safeguarded by elevating personal identity, a pillar of emotional well-being in person-centered dementia care. The care focus on maintaining identity is consistent with Kitwood’s person-centered framework (Kitwood & Bredin, 1992), and emphasizes the interconnectedness of a person’s body, mind, and soul in dementia care (Terkelsen et al., 2020). In this approach, ANs are pivotal in fostering compassionate care that constantly tries to acknowledge the resident’s sense of self. Caring for those who can no longer fully articulate their needs requires advanced skills and an understanding of shared lived experience, which Kitwood (1997) describes in person-centered care. Such an approach accounts for nurses’ interactions guided by the social, cognitive, and physical elements of dementia (Rose & Dening, 2023).

The *Theory of Validating Identity* is further supported by Watson (1985), who formulates that nursing experience symbolizes a multi-layered awareness that encompasses body, mind, and soul. Within this framework, the self is centered in its world, navigating its surroundings through freedom of choice and integrity, which are essential for fostering meaningful human interactions. The ability to create connections through emotional resonance and empathy is critical in caring for individuals who can no longer articulate their needs verbally and must rely on skilled nursing professionals. Profound self-reflection among nurses, as they contemplate their own emotions and identity connected to their caregiving, is essential for simulating meaningful care. (Dyring & Grøn, 2022; McGraw, 2002).

## Strengths and Limitations

CGT fits best when the final product—a theory grounded in the data—provides new conceptions and models, contributes to understanding fundamental social processes, and broadens generalizable relational development (Urquhart, 2008). As Glaser (1978) explains, the researcher should approach the field without prior knowledge and preconceived notions to engage effectively in the open categorization phase of CGT. This condition was particularly suitable for the data-collecting researcher, as she was unfamiliar with Swedish person-centered dementia care in nursing homes. The *Theory of Validating Identity* meets the CGT quality standards as it can be generalized and applied by nurses in other countries. The theory also remains flexible to accommodate the widening of categories and concepts for future use when more nursing care interventions and actions are identified. Furthermore, the generated theory should be clear and clinically applicable (Simmons, 2022). *The Theory of Validating Identity* illustrates this workability criterion, enabling nursing staff to utilize its practical applications effectively and implement them into their nursing care plans. The caring context, *Awakening Identity*, has three care interventions, but no care actions. However, it combines the three already known nursing tools—life story, reminiscence, and encountering BPSD—into care interventions that are comprehensively integrated into the theory dimensions. The *Shifting Orientation* care context encompasses several care interventions and actions, including newly discovered practices that were generated through the analytic process of addressing the core category.

The theory’s time dimension includes significantly fewer future nursing actions, since, due to the cognitive decline associated with dementia, nursing actions focused on the past and present are more effective in capturing residents’ time perspectives than those aimed at the future. In CGT, theoretical saturation occurs when newly identified properties no longer contribute new insights or variations to the emerging theoretical patterns but instead reaffirm what has already been established. The quality of a CGT study should convince readers that the identified categories and properties are appropriately connected to the core category and are consistent with the established substantive theory. According to CGT’s quality assurance, fit is a component of validity in which new information is derived during analysis, rather than relying on pre-existing data or established codes and categories within the theory. Furthermore, the theory should provide an abstract generalization of how the categories and properties are connected, allowing for modifications over time as more data becomes available (Glaser, 1998; Sandgren, 2010). *The Theory of Validating Identity* meets these quality criteria. To further develop the theory into a broader, mid-range theory, it should demonstrate that it extends beyond the original substantive setting by using additional samples, diverse care settings, and genders.

## Conclusion

The *Theory of Validating Identity* is a coherent complement to the person-centered dementia care framework. It can be applied especially for people who are suffering from behavioral and psychological symptoms of dementia. By purposefully applying the theory and its corresponding caring context, along with care interventions and actions, nurses can develop more sophisticated everyday nursing strategies. Implementing the theory in everyday care practice offers a way to strengthen care management, guide the development of comprehensive and individualized care plans, and refine existing care routines. It also has the potential to generate practical strategies for responding to residents’ confusion and alleviating staff distress.

## Appendix A

**Table A1.** Observation Mall, According to Spradley.*

Deltagare/Participant	Iakttagare/Observer	Boende/nursing home
Datum/Date	Annat/Other	Schema/Shift
Gruppmedlemmar/Team members	Observation börjar:/Observation begins: Observation slutar:/Observation ends:	Pacient/Patient
Beskriv utrymmen i detalj/Describe spaces in detail		
Beskriv föremålen/Discribe the objects		
Beskriv handling/Describe action		
Beskriv aktiviteten/Describe the activity		
Beskriv händelsen/Describe the event		
Beskriv tiden/Describe the time		
Beskriv aktören/Describe the actor/s		
Beskriv målet/Describe the goal		
Beskriv känslor/Describe feelings		

* in the original language and its English translation.
